# Autoimmune cerebellopyramidal syndrome as a complex form of autoimmune cerebellar ataxia: a cohort study

**DOI:** 10.3389/fimmu.2025.1724439

**Published:** 2025-12-08

**Authors:** Mange Liu, Haitao Ren, Siyuan Fan, Le Zhang, Hongzhi Guan

**Affiliations:** Department of Neurology, Peking Union Medical College Hospital, Beijing, China

**Keywords:** autoimmune cerebellar ataxia, pyramidal tract damage, autoimmune cerebellopyramidal syndrome, clinical features, prognosis

## Abstract

**Background:**

Autoimmune cerebellar ataxia (ACA) is a significant and treatable cause of sporadic cerebellar syndrome. Pyramidal tract damage affects approximately 30% of ACA patients, profoundly impacts motor function and prognosis, yet this complex form remains under-investigated.

**Methods:**

We retrospectively analyzed patients with pyramidal signs from our institutional ACA cohort, excluding those with intrinsic upper motor neuron impairment due to specific ACA subtypes. Clinical and paraclinical data were compared between patients with and without pyramidal signs.

**Results:**

Among the 48 ACA patients with pyramidal signs, primary autoimmune cerebellar ataxia was the most common subtype (50%), and neuronal antibodies were detected in 66.7%. Compared to ACA patients without pyramidal signs, those with pyramidal signs were younger at onset (44 *vs* 47 years, p=0.048) and more frequently presented with limb ataxia (97.9% *vs* 78.8%, p=0.002) and diplopia (48.9% *vs* 18.2%, p<0.001). Brain MRI revealed pyramidal tract and/or semiovale centrum lesions in 29.8% of patients with pyramidal signs, a finding absent in the non-pyramidal sign group (p<0.001), which could subside with immunotherapy. Cerebrospinal fluid (CSF) analysis showed higher protein concentration and a significantly higher rate of specific oligoclonal bands in the pyramidal sign group. Patients with pyramidal signs had a significantly higher risk of relapse (52.5% *vs* 32.8%, p=0.034).

**Conclusions:**

Autoimmune cerebellopyramidal syndrome represents a distinct and complex ACA phenotype, characterized by unique clinical and imaging features, CSF abnormalities indicative of a more pronounced autoimmune response, and a higher relapse rate. Comprehensive evaluation and long-term maintenance immunotherapy may be warranted in these patients.

## 1 Introduction

Autoimmune cerebellar ataxia (ACA) is an important and treatable cause of sporadic cerebellar syndrome. Based on the triggers of autoimmune process or the related anti-neuronal antibodies, ACA can be divided into several subtypes including paraneoplastic cerebellar degeneration (PCD), primary autoimmune cerebellar ataxia (PACA), glutamic acid decarboxylase (GAD) antibody-related cerebellar ataxia, metabotropic glutamate receptor 1 (mGluR1) antibody-related cerebellar ataxia, among others (1).

Although cerebellar syndrome is the main clinical presentation in ACA patients, extra-cerebellar involvement, particularly of the pyramidal tracts, is also frequent and can be observed in about 30% in ACA patients (2). This coexisting pyramidal tract involvement, which we term “autoimmune cerebellopyramidal syndrome,” introduces complexity to the diagnosis and assessment of ACA. Pyramidal tract damage can significantly impair a patient’s motor and coordination abilities, profoundly affecting their independence, quality of life, and overall prognosis (3). The presence of this cerebellopyramidal phenotype may also signify distinct pathogenetic mechanisms within ACA. However, dedicated studies focusing on the clinical features, anti-neuronal antibody profiles, and prognosis of ACA patients with pyramidal signs are mainly case studies and case series. This study aimed to analyze the characteristics and outcomes of patients presenting with this complex form of ACA in a relatively large cohort.

## 2 Methods

### 2.1 Participants

From 2013 to 2023, patients in Peking Union Medical College Hospital (PUMCH) were initially included in a broad ACA cohort if they had cerebellar syndrome as the main manifestation and met the diagnostic criteria of PCD (4), PACA (2), post infectious cerebellar ataxia (5) or GAD antibody-related cerebellar ataxia (6). This broader cohort also included patients with other neuroimmune diseases including opsoclonus-myoclonus syndrome (OMS), Miller Fisher syndrome (MFS), Bickerstaff brainstem encephalitis (BBE), and autoimmune encephalitis if cerebellar ataxia was the predominant or one of the major symptoms (2, 4, 6). Central nervous system demyelinating diseases including multiple sclerosis, neuromyelitis optica spectrum disorder and myelin oligodendrocyte glycoprotein antibody-associated disease were excluded from this initial cohort. Patients of all ages were eligible. The detailed protocol for patients’ recruitment, clinical evaluation and follow-up procedures has been described previously (2, 7). Screening for infection, toxins, metabolic and hereditary diseases were also completed if clinically appropriate to exclude other causes of ataxia. Serum and cerebrospinal fluid (CSF) ACA antibody panel (Tr(DNER)/Zic4/ITPR1/Homer-3/neurochondrin/PKCγ/PCA-2/AP3B2/mGluR1/ATP1A3/CARPVIII/Sez6L2/ARHGAP 26 antibodies) was examined using cell-based assay (CBA) and tissue-based assay (TBA) (EUROIMMUN, Lübeck, Germany). Rab6 antibody and EEF1D antibody were identified at our institution and thus were assessed by TBA and an in-house CBA (8, 9). Paraneoplastic antibody assays (Hu/Yo/Ri/Ma2/Ta/CV2/amphiphysin antibodies, immunoblot, EUROIMMUN, Lübeck, Germany) and autoimmune encephalitis antibodies (NMDAR/LGI1/GABAR/CASPR2/GAD65/IgLON5/DPPX antibodies, CBA and TBA, EUROIMMUN, Lübeck, Germany) were also examined.

For the purpose of the current comparative study, we specifically screened and included patients with pyramidal signs from this PUMCH ACA cohort. Pyramidal signs were defined as the presence of unilateral or bilateral hyperreflexia, pathological reflexes, spasticity, or clonus, with or without pyramidal weakness, at the time of enrollment or subsequent follow-up. This study also compares ACA patients with and without pyramidal signs (PS group and non-PS group).

To ensure that the pyramidal signs analyzed were not intrinsically defined features of other well-established neurological autoimmune conditions, certain ACA subtypes were excluded from this specific analysis. Specifically, patients with anti-GAD associated ataxia (n=15), anti-CV2 associated ataxia (n=4), and BBE (n=2) were excluded because upper motor neuron impairment is an intrinsic and well-established feature of these conditions (10, 11). MFS cases were also excluded from this comparative analysis, as MFS is characterized by ophthalmoplegia, ataxia, and areflexia, and by definition typically lacks pyramidal signs (12). Furthermore, patients with other conditions capable of causing pyramidal signs, such as spinal cord compression, myelopathy, metabolic diseases, or a history of cerebrovascular disease, were carefully excluded (n=9) after comprehensive clinical evaluation and neuroimaging. An additional 20 patients were excluded due to insufficient data to determine the presence or etiology of pyramidal tract damage.

Informed consent was obtained from all individual participants or their deputies included in the study. This study was performed in line with the principles of the Declaration of Helsinki. Approval was granted by the Ethics Committee of Peking Union Medical College Hospital (JS-891).

### 2.2 Data analysis

All continuous data were expressed as medians with interquartile range (IQR) due to non-normal distribution. Categorical data were expressed as frequencies with percentage. The Mann–Whitney test was used for intergroup comparisons of continuous variables. The Pearson χ2 test or Fisher’s exact test was used for categorical variables. Statistical analyses were conducted using SPSS software version 26.0 (IBM Corp., Armonk, NY, USA). A p-value < 0.05 was considered significant.

## 3 Results

### 3.1 Clinical characteristics

A total of 48 ACA patients with definite pyramidal signs were included in the study, comprising 17 males (35.4%). The median age at symptom onset was 44 (IQR 23.8-50.8) years. Most patients experienced subacute (symptom progression for 2 weeks to 3 months, n=35, 76.1%) or acute (symptom progression within 2 weeks, n=8, 17.4%) symptom onset. The most common symptom was gait ataxia (*n* =46, 95.8%) and limb ataxia (*n* =46, 95.8%), followed by dysarthria/dysphagia (*n* =37, 77.1%), dizziness (*n* =30, 62.5%), diplopia (*n* =23, 47.9%) and nystagmus (*n* =20, 41.7%).

The distribution of pyramidal signs was as follows: 14 patients (29.2%) with pyramidal weakness, 8 (16.7%) with spasticity, 19 (39.6%) with hyperreflexia, and 45 (93.8%) with pathological reflexes. Pyramidal tract signs were unilateral in only 2 (4.2%) patients, while the remaining 46 (95.8%) exhibited bilateral involvement. Among the 8 patients with spasticity, Modified Ashworth Scale scores were 1 in 5 patients, 2 in 2 patients, and 3 in 1 patient. Regarding the 14 patients with limb weakness, 3 had a minimum muscle strength of Grade 3 (Medical Research Council scale), and 11 had a minimum strength of Grade 4. In addition to pyramidal signs, extra-cerebellar involvement also presented as peripheral neuropathy/radiculopathy in 9 patients (18.8%). Besides, fifteen (31.3%) patients experienced prodromal infection-like symptoms. Malignancy was identified in 10 (20.8%; lung cancer in 3, ovary cancer in 2, breast cancer in 3, and other types of cancer in 2) patients.

### 3.2 Laboratory and magnetic resonance imaging characteristics

**Figure 1:**
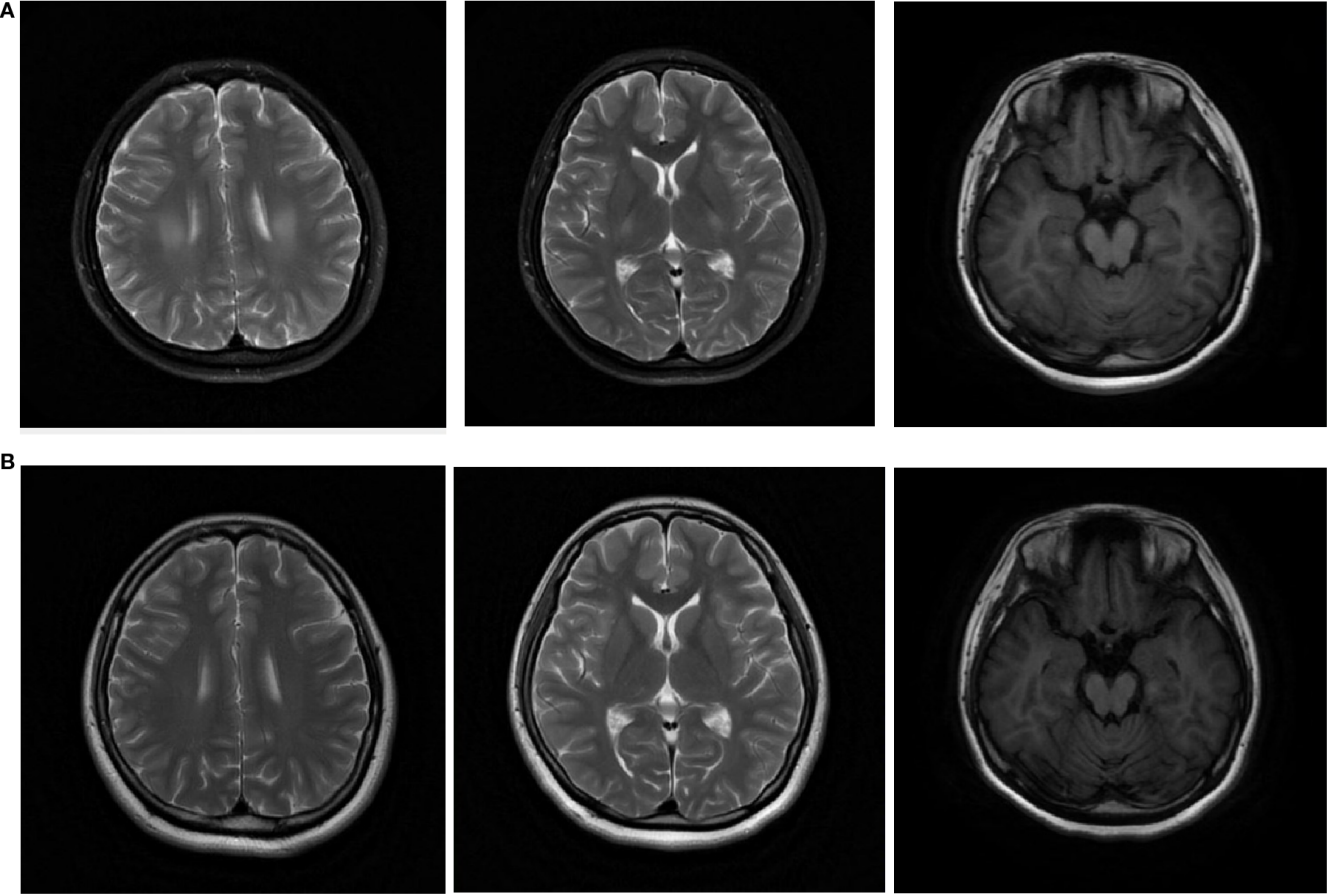
Brain MRI of a patient showed lesions in bilateral pyramidal tracts and semiovale centrum at disease onset **(a)**. In the follow-up MRI conducted 17 months later, the lesions significantly improved while cerebellar atrophy were noticed **(b)**. Left and middle: T2 sequence; right: T1 sequence.

Examination of systemic autoantibodies revealed that 9 (20%) patients were positive for anti-SSA/SSB antibodies. Seven patients (15.5%), including two with Hashimoto’s thyroiditis, were positive for anti-thyroid peroxidase (TPO) and/or thyroglobulin (Tg) antibodies. An additional 4 patients (8.9%) tested positive for both anti-SSA/SSB and anti-TPO/Tg antibodies. Another 5 (11.1%) patients were anti-nuclear antibody (ANA)-positive but anti-SSA/SSB-negative. Six patients were diagnosed with Sjogren’s syndrome. The first brain magnetic resonance imaging (MRI) scan available in the disease course showed T2 hypersensitivity lesions in 20 patients (42.6%), specifically located in the bilateral pyramidal tracts (n=11), semiovale centrum (n=3), cerebellum (n=2), and other sites (n=4). Cerebellar atrophy was observed in 7 patients (14.9%). In 20 patients (42.6%), the results of brain MRI were normal. The pyramidal tract lesions could subside after immunotherapy, and cerebellar atrophy might appear in follow-up (**Figure 1**). For CSF examinations, the median CSF white blood cell count and protein concentration were 8 (IQR 2-23.8)/μL and 0.58 (IQR 0.42-1.06) g/L, respectively. CSF pleocytosis (WBC count>5/μL) was detected in 28 patients (range 6-380/μL). CSF-specific oligoclonal bands were identified in 34 (87.2%) patients.

### 3.3 Neuronal antibodies and nosology

Regarding the distribution of ACA subtypes in the PS-group, PACA was the most common (n=24, 50%), followed by PCD (n=7, 14.6%). Overall, neuronal antibodies were identified in 32 (66.7%) patients, as shown in **Table 1a**. ACA subtypes and neuronal antibodies for the non-PS group are presented in **Table 1b**.

**Table 1a:** Distribution of ACA subtypes and neuronal antibodies in patients with pyramidal signs.

ACA subtype	Neuronal antibodies	No	ACA subtype	Neuronal antibodies	No
PACA		24	PCD		7
	TBA+	8		Yo	4
	Homer3	2		Ri	2
	Rab6	1		Hu&SOX	1
	Sez6L2	1	PIC		5
	ARHGAP26	1		TBA+	2
	Neuronal Ab-negative	11		Neuronal Ab-negative	3
OMS		3	AE-ACA		5
	Amphiphysin	1		NMDAR	3
	Neuronal Ab-negative	2		Caspr2	1
mGLuR1	mGLuR1	4		DPPX	1

**Table 1b:** Distribution of ACA subtypes and neuronal antibodies in patients without pyramidal signs.

ACA subtype	Neuronal antibodies	No	ACA subtype	Neuronal antibodies	No
PACA		57	PCD		47
	TBA	12		Yo	14
	Homer3	6		Hu	11
	NCDN	3		Tr	7
	Sez6L2	2		SOX1	4
	EEF1D	2		Amphiphysin	2
	ARHGAP26	1		TBA	1
	Zic4	1		Zic4	1
	PCA2	1		PCA2	1
	AP3B2	1		Ma2	1
	CARP8	1		Neuronal Ab-negative	5
	Neuronal Ab-negative	27	AE-ACA		14
OMS		2		NMDAR	11
	TBA	1		GABAR	1
	Neuronal Ab-negative	1		DPPX	1
PIC		6		IgLON5	1
	TBA+	1	mGLuR1	mGLuR1	6
	Neuronal Ab-negative	5			

*ACA* autoimmune cerebellar ataxia; *AE* autoimmune encephalitis; *OMS* opsoclonus-myoclonus syndrome; *PACA* primary autoimmune cerebellar ataxia; *PCD* paraneoplastic cerebellar degeneration; *PIC* post infectious cerebellar ataxia.

### 3.4 Treatment and prognosis

For first-line immunotherapy, 35 (72.9%) patients received a combination of intravenous immunoglobulin (IVIg) and corticosteroids. Six (12.5%) patients received IVIg alone, and 7 patients (14.6%) received corticosteroids alone. A total of 37 (82.2%) patients demonstrated clinical improvement following first-line immunotherapy. Long-term immunosuppressive agents were prescribed to a subset of patients, including mycophenolate mofetil (n=21), rituximab (n=16), and other agents (n=6). After a median follow-up period of 19 (IQR 10-24) months from disease onset, the median modified Rankin Scale (mRS) score was 2 (IQR 1–4) and the median Scale for the Assessment and Rating of Ataxia (SARA) score was 12 (IQR 7–19). A favorable prognosis, defined as an mRS score ≤2 at the last follow-up, was achieved by 25 patients (53.2%). Sixteen (33.3%) patients relapsed.

### 3.5 Comparison between ACA patients with and without pyramidal signs

A comparative analysis between the pyramidal signs (PS) group (n=48) and the non-pyramidal signs (non-PS) group (n=132) revealed several significant differences. Patients in the PS group were younger at disease onset (median 44 years [IQR 23.8–50.8] *vs* 47 years [IQR 34.5–59], p=0.048). They also showed a higher prevalence of PACA, post-infectious cerebellar ataxia (PIC), or anti-mGluR1 antibody-associated cerebellar ataxia (details in **Table 2**). Neurological symptoms such as limb ataxia (97.9% *vs* 78.8%, p=0.002) and diplopia (48.9% *vs* 18.2%, p<0.001) were significantly more frequent in the PS group. Brain MRI examination detected lesions in the pyramidal tracts and/or semiovale centrum in 29.8% of the PS group, a finding entirely absent in the non-PS group (p<0.001). Furthermore, the PS group exhibited a higher median CSF protein concentration (0.58 g/L [IQR 0.42–1.06] *vs* 0.46 g/L [IQR 0.32–0.68], p=0.003) and a significantly higher prevalence of CSF-specific oligoclonal bands (SOB) (87.2% *vs* 51.1%, p<0.001). Although mRS score at the last follow-up were comparable between the two groups (2 (1, 4) *vs* 3 (1, 4), p=0.526), the risk of relapse was significantly higher in the PS group (52.5% *vs* 32.8%, p=0.034).

**Table 2:** Comparison between ACA patients with and without pyramidal signs.

Variables	ACA without pyramidal signs (n=132)	ACA with pyramidal signs (n=48)	P value
Male/no. (%)	63 (47.7)	17 (35.4)	0.142
Age at onset/years	47 (34.5, 59)	44 (23.8, 50.8)	**0.048**
ACA subtypes			**0.027**
PCD/no. (%)	47 (35.6)	7 (14.6)	
PACA/no. (%)	57 (43.2)	24 (50)	
PIC/no. (%)	6 (4.5)	5 (10.4)	
OMS/no. (%)	2 (1.5)	3 (6.3)	
AE-ACA/no. (%)	14 (10.6)	5 (10.4)	
Anti-mGLuR1 ACA/no. (%)	6 (4.5)	4 (8.3)	
Neurological symptoms			
Limb ataxia/no. (%)	104 (78.8)	46 (97.9)	**0.002**
Diplopia/no. (%)	24 (18.2)	23 (48.9)	**<0.001**
Brain MRI			**<0.001**
Unremarkable/no. (%)	80 (63.5)	20 (42.6)	
Atrophy/no. (%)	26 (20.7)	7 (14.9)	
PT or SO lesions/no. (%)	0 (0)	14 (29.8)	
Lesions in other parts/no. (%)	20 (15.9)	6 (12.8)	
CSF examinations			
WBC count/μL^-1^	5.5 (2, 23)	8 (2, 23.3)	0.573
Protein concentration/g·L^-1^	0.46 (0.32, 0.68)	0.58 (0.42, 1.06)	**0.003**
CSF SOB/no. (%)	45 (51.1)	34 (87.2)	**<0.001**
Immunotherapy			
Corticosteroids pulse/no. (%)	65 (53.3)	30 (68.2)	0.087
Oral corticosteroids/no. (%)	56 (48.7)	16 (44.4)	0.656
IVIg/no. (%)	89 (72.4)	41 (89.1)	**0.021**
MMF/no. (%)	38 (32.8)	21 (52.5)	**0.026**
Relapse/no. (%)	25 (20.3)	16 (36.4)	**0.034**
mRS at zenith	4 (2, 4)	4 (2, 4)	0.728
mRS score at last follow-up	3 (1, 4)	2 (1, 4)	0.526

*AE* autoimmune encephalitis; *CSF* cerebrospinal fluid; *IVIg* intravenous immunoglobulin; *MMF* mycophenolate mofetil; *mRS* modified Rankin scale; MRI magnetic resonance imaging; *OMS* opsoclonus-myoclonus syndrome; *PACA* primary autoimmune cerebellar ataxia; *PCD* paraneoplastic cerebellar degeneration; *PIC* post infectious cerebellar ataxia; *PT* pyramidal tracts; *SO* semiovale centrum; *SOB* (CSF)-specific oligoclonal bands; *WBC* white blood cell. Bold text indicates statistical significance and a p value < 0.05.

## 4 Discussion

Our study provides a detailed analysis of the clinical and paraclinical features of ACA patients presenting with pyramidal signs, conceptualizing this as a complex form of ACA. Key findings include: 1) PACA being the predominant subtype among ACA patients with pyramidal signs; 2) Pyramidal tract and semiovale centrum hyperintensity on brain MRI serving as a specific imaging marker for pyramidal involvement, observed in approximately 30% of these patients; and 3) Compared to ACA patients without pyramidal signs, those with this complex form of ACA were younger at onset and demonstrated a higher propensity for relapse, although the p-value of 0.048 when comparing the age at onset, which was close to 0.05, indicated that the reliability and clinical significance of this difference needed to be verified in a larger cohort.

Current diagnostic criteria for primary autoimmune cerebellar ataxia (PACA), as outlined by the International Task Force in 2020, primarily emphasize a pure cerebellar syndrome (13). However, accumulating evidence from previous studies and our institutional experience suggests a broader spectrum of neurological involvement in both PACA and general ACA patients. For instance, a study by McKeon et al. reported pyramidal tract signs in 30% of 83 patients seropositive for Yo antibody (14). Similarly, in a large cohort of patients with anti–Ri-associated paraneoplastic neurological syndrome,13 out of 36 patients (36%) developed pyramidal hypertonia (15). Pyramidal tract signs have also been documented in ACA patients mimicking multiple system atrophy (16, 17). In our ACA cohort, pyramidal tract signs were observed in 28.3% of patients (2). These observations suggest that strictly limiting the diagnosis of ACA and PACA to those with pure cerebellar symptoms might lead to missed diagnoses and challenges in clinical management. Consequently, based on these findings, we have previously expanded the definition and proposed modified diagnostic criteria for PACA to encompass patients with extra-cerebellar symptoms, including pyramidal signs (2, 7). In the present study, we further characterized ACA patients with pyramidal signs, defining this as a complex form of ACA. We propose that the presence of pyramidal signs should not be considered a contraindication or ‘red flag’ against an ACA diagnosis.

In our study, T2 hyperintensity in the pyramidal tracts or semiovale centrum was observed in 29.8% of ACA patients with pyramidal signs, a finding absent in ACA patients without pyramidal signs. This suggests that such MRI features are specific imaging markers for clinical pyramidal tract damage. Previous literature has reported cerebrospinal tract hyperintensity on brain MRI in various neurological autoimmune diseases, such as those associated with anti-LGI1, anti-Ma2, anti-GAD65 and anti-GFAP antibodies, as well as demyelinating diseases including neuromyelitis optica and myelin oligodendrocyte glycoprotein-associated disease (18) (19, 20). Consistent with our findings, these pyramidal tract abnormalities can also attenuated after immunotherapy. Our ongoing research involves performing diffusion tensor imaging in ACA patients with pyramidal signs to identify a more sensitive imaging modality.

The precise mechanism underlying pyramidal tract injury in ACA patients remains unclear. However, the significantly higher rate of CSF SOB positivity in ACA patients with pyramidal tract damage suggests a potential generalization of the autoimmune response within the central nervous system in this subset of patients. A similar phenomenon has been reported in myasthenia gravis patients, where the CSF IgG synthetic rate was higher in those with pyramidal signs compared to those without central nervous system involvement (21). Besides, the higher proportions of patients with diplopia and limb ataxia in the PS-group may suggest broader and more pronounced disease involvement encompassing the brainstem and cerebellar hemispheres, as previous studies have demonstrated brainstem infiltrating of cytotoxic T-lymphocytes in ACA patients (22).

Recently, anti-vimentin antibodies have been reported to be associated with cerebellar ataxia and pyramidal tract damage (23). However, our study suggested that cerebellar ataxia with pyramidal tract involvement can be associated with various antineuronal antibodies. In fact, among our patients in the PS-group, three were tested for anti-vimentin antibodies, and all results were negative. In our cohort, 22 patients tested positive for known anti-neuronal antibodies, while an additional 10 were positive in a tissue-based assay (TBA) but lacked identified antibodies via cell-based assay (CBA). It is noteworthy that many target molecules of these known autoantibodies exhibit low brain region specificity and are expressed in areas such as the precentral gyrus and white matter (e.g., NMDAR, mGluR1, as per https://www.proteinatlas.org/). The exact contribution of these autoantibodies to the pathogenesis of pyramidal tract damage, or whether they merely represent bystanders of the autoimmune process, warrants further investigation.

ACA patients with pyramidal signs exhibited a significantly higher risk of relapse. This is probably related to the more active autoimmune process in these patients, as suggested by the higher rate of CSF-SOB. Similarly, in multiple sclerosis, oligoclonal band positivity has been correlated with more spinal lesions on MRI and increased relapse rates (24). Thus, it is reasonable to perform comprehensive CSF analysis and consider prolonged course of immunotherapy in ACA patients with pyramidal signs.

This study has several limitations. Firstly, while we observed significant differences in clinical and paraclinical features and prognosis in ACA patients with pyramidal signs, the underlying mechanism of pyramidal tract damage remains unclear, and we did not identify specific autoantibodies solely responsible for these signs. Therefore, whether ACA patients with pyramidal signs constitute a truly independent disease entity warrants further investigation. Secondly, the assessment of pyramidal tract damage primarily relied on physical examination. Future studies incorporating advanced neuroimaging techniques, such as diffusion tensor imaging, could prove beneficial in detecting subtle or subclinical pyramidal tract damage that might have been overlooked in the current study.

In conclusion, our study indicates that acute/subacute onset cerebellopyramidal syndrome is a complex and distinct form of ACA, characterized by unique clinical and paraclinical features. Comprehensive diagnostic testing, including lumbar puncture and anti-neuronal antibody screening, is reasonable, as prompt treatment can facilitate improved patient outcomes. Furthermore, given the significant association between pyramidal signs and a higher risk of relapse, long-term maintenance immunotherapy may be crucial for optimizing prognosis in these patients.

## Data Availability

The raw data supporting the conclusions of this article will be made available by the authors, without undue reservation.
